# The role of meteorological factors on influenza incidence among children in Guangzhou China, 2019–2022

**DOI:** 10.3389/fpubh.2023.1268073

**Published:** 2024-01-08

**Authors:** Zhitao Chen, Yanhui Liu, Haiyan Yue, Jinbin Chen, Xiangzhi Hu, Lijuan Zhou, Boheng Liang, Guozhen Lin, Pengzhe Qin, Wenru Feng, Dedong Wang, Di Wu

**Affiliations:** ^1^Department of Public Health and Preventive Medicine, School of Medicine, Jinan University, Guangzhou, China; ^2^Guangzhou Center for Disease Control and Prevention, Guangzhou, China; ^3^School of Public Health, Institute of Public Health, Guangzhou Medical University and Guangzhou Center for Disease Control and Prevention, Guangzhou, China; ^4^Guangzhou Meteorological Observatory, Guangzhou, China; ^5^Guangzhou Key Laboratory for Clinical Rapid Diagnosis and Early Warning of Infectious Diseases, KingMed School of Laboratory Medicine, Guangzhou Medical University, Guangzhou, Guangdong, China

**Keywords:** meteorological factors, influenza, distributed lag non-linear models, children, incidence

## Abstract

**Objective:**

Analyzing the epidemiological characteristics of influenza cases among children aged 0–17 years in Guangzhou from 2019 to 2022. Assessing the relationships between multiple meteorological factors and influenza, improving the early warning systems for influenza, and providing a scientific basis for influenza prevention and control measures.

**Methods:**

The influenza data were obtained from the Chinese Center for Disease Control and Prevention. Meteorological data were provided by Guangdong Meteorological Service. Spearman correlation analysis was conducted to examine the relevance between meteorological factors and the number of influenza cases. Distributed lag non-linear models (DLNM) were used to explore the effects of meteorological factors on influenza incidence.

**Results:**

The relationship between mean temperature, rainfall, sunshine hours, and influenza cases presented a wavy pattern. The correlation between relative humidity and influenza cases was illustrated by a U-shaped curve. When the temperature dropped below 13°C, Relative risk (RR) increased sharply with decreasing temperature, peaking at 5.7°C with an RR of 83.78 (95% CI: 25.52, 275.09). The RR was increased when the relative humidity was below 66% or above 79%, and the highest RR was 7.50 (95% CI: 22.92, 19.25) at 99%. The RR was increased exponentially when the rainfall exceeded 1,625 mm, reaching a maximum value of 2566.29 (95% CI: 21.85, 3558574.07) at the highest rainfall levels. Both low and high sunshine hours were associated with reduced incidence of influenza, and the lowest RR was 0.20 (95% CI: 20.08, 0.49) at 9.4 h. No significant difference of the meteorological factors on influenza was observed between males and females. The impacts of cumulative extreme low temperature and low relative humidity on influenza among children aged 0–3 presented protective effects and the 0–3 years group had the lowest RRs of cumulative extreme high relative humidity and rainfall. The highest RRs of cumulative extreme effect of all meteorological factors (expect sunshine hours) were observed in the 7–12 years group.

**Conclusion:**

Temperature, relative humidity, rainfall, and sunshine hours can be used as important predictors of influenza in children to improve the early warning system of influenza. Extreme weather reduces the risk of influenza in the age group of 0–3 years, but significantly increases the risk for those aged 7–12 years.

## 1 Introduction

Influenza is an acute febrile respiratory infectious disease caused by influenza viruses (1). The World Health Organization estimates that the annual influenza epidemic results in approximately 3–5 million cases of severe illness and 290,000 to 650,000 influenza-associated respiratory deaths worldwide (2). Children under 59 months are at a higher risk of severe influenza or complications. Approximately 10 to 15% of the global population is infected with influenza viruses every year (3). The incidence of influenza is about 5 to 10% in adults and up to 20 to 30% in children. The positive rate of influenza tests varies among different age groups, with children aged 5–15 years having the highest positive rate (4). It is estimated that the mortality rate among children under 5 years of age with influenza-related lower respiratory tract infections is as high as 99% in developing countries and about 9,243 to 105,690 children under 5 years of age die from influenza-associated respiratory disease annually in 92 countries (5, 6). In addition, studies have demonstrated that influenza markedly increases the number of outpatient visits for infants and children (7), with an average of 90 million new cases of influenza among children younger than 5 years and generally 870,000 children under 5 years of age being admitted to hospitals for influenza annually (5, 8). The epidemics of influenza impose a substantial medical and economic burden on global health systems and societies, and pose a severe threat to the lives and health of children.

Previous studies have demonstrated that meteorological factors play an important role in influenza transmission, but these factors may have different effects on the spread of influenza in different regions (9–12), highlighting the complexity and diversity in the relationship between climate factors and influenza transmission. Temperature and humidity are considered as the most crucial meteorological indicators influencing influenza epidemics (13, 14). Generally, lower temperatures and humidity increase the risk of influenza. In temperate regions, influenza exhibits distinct seasonality, with peak activity during winter (December to March in the northern hemisphere and May to September in the southern hemisphere). This is because cold and dry environments facilitate the survival and migration of the influenza virus (15, 16). In tropical and subtropical regions, seasonal influenza is more varied, with prevalent activity throughout the year and two peaks in both winter and summer (17). It’s suggested that influenza epidemics are closely related to rainy seasons and increased rainfall (18), but further researches are needed. Furthermore, other climatic factors such as wind speed, atmospheric pressure, and sunlight duration are also considered to be associated with influenza activity (10, 11, 19), although the correlation is generally small or not statistically significant. The combined effects of these meteorological factors contribute to the disparities in influenza prevalence across different temperature zones. Therefore, no definitive consensus exists on the relationship between meteorological factors and influenza. Further exploration is required to understand he correlation between meteorological factors and influenza epidemic. This study aims to examine the relationship between multiple meteorological factors and pediatric influenza in Guangzhou, a subtropical city.

Understanding the role of meteorological factors in influenza epidemics and discerning the patterns of these epidemics can significantly aid in the implementation of effective control strategies. In this study, we first analyzed the epidemiological characteristics of influenza cases among children aged 0–17 years in Guangzhou from 2019 to 2022. Then, distributed lag nonlinear model (DLNM) with the daily series data was used to evaluate the relationships between various meteorological factors and influenza. The findings from this study can contribute to the development of early warning systems for influenza, providing practical and reliable scientific basis for implementing influenza prevention and control measures, thus reducing the medical and economic burden associated with influenza in children.

## 2 Methods

### 2.1 Study area

Guangzhou, situated in south China, is the capital of Guangdong Province. It spans from 112°57′E to 114°3′E and 22°26′N to 23°56′N. With a total area of 7434.4 square kilometers, Guangzhou is divided into 11 administrative districts, and its resident population reached 18.81 million by 2021. The city experiences a subtropical monsoon climate, with a notable influence from the marine climate. This climate is characterized by long, hot, and rainy summers, as well as short, mild, and dry winters.

### 2.2 Data collection

The influenza data collected in this study from 1 January 2019 to 31 December 2022, were obtained from the National Notifiable Disease Surveillance System of the Chinese Center for Disease Control and Prevention. The dataset includes individual case data, consisting of case identification number, demographic information (gender, age, and the type of childcare), onset, and diagnosis times, case classifications, and reporting authorities.

Influenza is classified as a Class “C” infectious disease in China and must be reported to the National Disease Prevention and Control Information System within 24 h of diagnosis. According to the Influenza Diagnosis and Treatment Protocol (2020 Edition), influenza cases can be categorized as suspected cases, clinically diagnosed cases, or confirmed cases. Suspected cases are those with clinical flu symptoms and either an epidemiological history or positive auxiliary examinations. Clinically diagnosed cases refer to suspected cases in which other diseases causing similar symptoms have been ruled out. Confirmed cases are individuals with clinical flu symptoms and one or more positive results from pathogenic testing. In this study, we included clinically diagnosed and confirmed influenza cases, while excluding suspected cases.

The meteorological data used in this study were provided by Guangdong Meteorological Service.^1^ The climate factors included daily average temperature (°C), maximum temperature (°C), minimum temperature (°C), average relative humidity (mm), average wind speed (m/s), atmospheric pressure (hpa), average rainfall (mm), and sunshine hours (h). The daily temperature range (DTR) was calculated by subtracting the daily minimum temperature from the maximum temperature. Only a small number of missing values were present, and these were resolved using multiple imputation.

### 2.3 Statistical analysis

We first conducted a descriptive analysis of the meteorological data and influenza data for children in Guangzhou and plotted a scatter plot to observe the relationship between meteorological factors and the daily number of influenza cases. Based on the nonlinear relationship observed in the scatter plot and the lagged effects of meteorological factors on health, DLNMs were used to explore the impact of meteorological factors on influenza incidence. This model can take into account the nonlinear exposure-response relationship and the lag effect of exposure factors on outcomes simultaneously (20). It has been widely used to evaluate the expose-lag-effect correlations between meteorological factors and infectious diseases, such as scarlet fever, mumps, and acute respiratory infections (21–23). Spearman correlation analysis was conducted as a preliminary analysis to examine the relevance between climate factors and the number of influenza cases. Considering the overdispersion of daily influenza cases, a Poisson regression with quasi-Poisson function was established to construct the model. The DLNMs structure was as follows:


$$\begin{array}{l} \log\left[E\left(Y_{t}\right)\right]=a+cb\left(x_{i},lag,df\right)+\sum ns\left(x_{j},df\right)+\\ \;\;\;\;\;\;\;\;\;\;\;\;\;\;\;\;\;\;\;\;\;\;\;\;\;\;\;\;\;\;\;\;\;ns\left(time,df*4\right)+\delta \left(dow\right)+\delta \left(holiday\right) \end{array}$$

where *Y_t_* represented the daily number of influenza cases on day *t*; a was the intercept; cb represented the cross-basis matrix of meteorological factors, which using natural cubic spline function or natural cubic B-spline function; ns() was a natural cubic spline function; *x_i_* was one of the meteorological variables, such as mean temperature, relative humidity, rainfall, and sunshine hours; *x_j_* represented the climate variables other than *x_i_*; time denoted long term trends and seasonality; dow denoted the day of week effect; holiday referred to a binary variable for control for summer and winter vacations as well as all Chinese legal vacations; df was degree of freedom. According to Akaike information criterion (AIC) and references (10, 11, 24), we chose *df* = 3 for *x_j_*, *df* = 6 for time, *df* = 5 for mean temperature, relative humidity and sunshine hours, and *df* = 3 for rainfall. After taking into account incubation period of influenza, lag = 4 weeks was chosen for meteorological factors based on the AIC and other references (10, 19).

We calculated the extreme high effects by comparing the 97.5th percentiles to the median values, and the extreme low effects by comparing the 2.5th percentiles to the median values. Relative risk (RR) with corresponding 95% confidence interval was used to estimate these effects. The cumulative relative risks were further explored. Moreover, we estimated the effects of meteorological on influenza by gender and age group.

All statistical tests were two-sided and *P* < 0.05 was considered statistically significant. All analyses were performed using the “mice,” “Hmisc” and “dlnm” packages in R (version 4.2.1).

Sensitivity analyses were performed by adjusting df per year within the range of 4–7 to account for long-term trends and seasonality. Additionally, the df for mean temperature, relative humidity, and sunshine hours were adjusted within the range of 2–5. Furthermore, we changed the maximum lag weeks (2–4 weeks) for meteorological factors. Since there was no rainfall on most days, changing the degree of freedom for rainfall would lead to errors in the model. Therefore, only the lag weeks for rainfall were changed.

## 3 Results

### 3.1 Descriptive statistics for general characteristics

**Figure 1:**
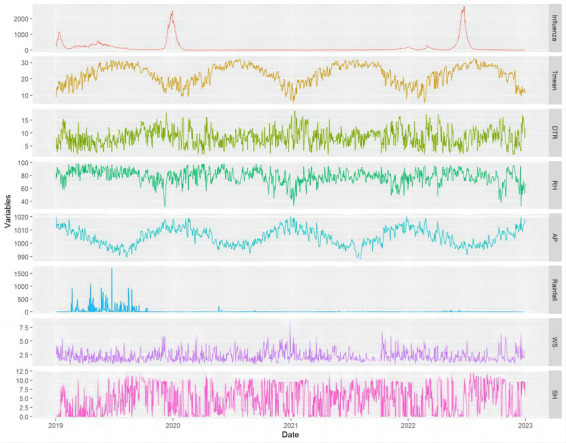
The distribution of daily influenza cases of children and meteorological variables in Guangzhou, China, 2019–2022. Tmean, mean temperature; DTR, daily temperature range; RH, mean relative humidity; AP, mean atmospheric pressure; Rainfall, aggregate rainfall; WS, mean wind speed; SH, sunshine hours.

From 1 January 2019 to 31 December 2022, a total of 210,835 clinically or laboratory confirmed cases of influenza among children aged 0–17 years were reported in Guangzhou. Among these cases, 122,077 (57.90%) were males and 88,758 (42.10%) were females. The number of influenza cases of children aged 0–3, 4–6, 7–12 and 13–17 was 65,561 (31.10%), 56,772 (26.93%), 68,503 (32.49%) and 19,999 (9.49%), respectively. The yearly breakdown of influenza cases from 2019 to 2022 was as follows: 106,426 (50.48%) in 2019, 23,902 (11.34%) in 2020, 7,323 (3.47%) in 2021, and 73,184 (34.71%) in 2022. The maximum and minimum number of daily influenza cases was 2,793 and 0, respectively, with a mean (standard deviation) of 144.3 (365.7). In terms of time series, the influenza activity showed a wave-like pattern in 2019, with two epidemic peaks observed during the winter of 2019–2020 and the summer of 2022. The number of influenza cases maintained at an extremely low level from spring 2020 to autumn 2021, which may be related to the nationwide outbreak of Corona Virus Disease 2019 (COVID-19; Figure 1; Table 1).

**Table 1:** Descriptive statistics of daily influenza cases of children and meteorological variables in Guangzhou, China, 2019–2022.

Variables	Mean	SD	Min	P25	P50	P75	Max
Influenza	144.30	365.70	0	6	14	113	2,793
Male	83.56	211.58	0	4	9	65	1,664
Female	60.75	154.46	0	2	6	47	1,129
0–3 years	44.87	99.11	0	3	7	27	826
4–6 years	38.86	103.86	0	1	4	24	914
7–12 years	46.89	136.49	0	1	3	32	1,149
13–17 years	13.69	42.04	0	0	1	7	351
Tmean (°C)	22.69	5.89	5.70	18.20	23.60	27.70	32.30
Tmax (°C)	27.76	6.05	6.90	23.50	28.80	32.80	38.10
Tmin (°C)	19.28	6.09	1.10	14.40	20.30	24.60	29.50
DTR (°C)	8.48	3.21	1.60	6.20	8.30	10.60	18.50
RH (%)	78.42	10.87	31.00	72.50	79.00	86.00	99.00
AP (hpa)	1004.90	6.42	987.80	999.60	1004.9	1009.8	1020.8
Rainfall (mm)	20.55	94.10	0.00	0.00	0.00	3.60	1718.00
WS (m/s)	2.27	0.98	0.80	1.60	2.00	2.80	8.80
SH (h/d)	4.79	3.81	0.00	0.70	4.80	8.40	12.10

SD, standard deviation; Influenza, all influenza cases; Male, number of male cases; Female, number of female cases; 0–3 years, number of cases aged 0–3 years; 4–6 years, number of cases aged 4–6 years; 7–12 years, number of cases aged 7–12 years; 13–17 years, number of cases aged 13–17 years; Tmean, mean temperature; Tmax, maximum temperature; Tmin, minimum temperature; DTR, daily temperature range; RH, mean relative humidity; AP, mean atmospheric pressure; Rainfall, aggregate rainfall; WS, mean wind speed; SH, sunshine hours.

Daily meteorological data were shown in Table 1 and Figure 1. The maximum and minimum temperature was 38.10°C and 1.10°C, respectively. The average daily temperature, daily temperature range, relative humidity, atmospheric pressure, rainfall, wind speed, and sunshine hours were 22.69°C, 8.48°C, 78.42%, 1004.90 hpa, 20.55 mm, 2.27 m/s, and 4.79 h/d, respectively. The average temperature, relative humidity, atmospheric pressure displayed seasonal patterns, with higher temperatures and relative humidity in the summer and lower values in the winter. In contrast, atmospheric pressure showed the opposite trend. Aggregate rainfall was higher in 2019 but remained extremely low from 2020 to 2022. Throughout this three-year period, there were a total of 656 days (59.8%) with no rainfall recorded.

### 3.2 Spearman correlation analysis

Table 2 provided the matrix of Spearman correlation coefficients between pediatric influenza cases and meteorological variables. Mean temperature and sunshine hours exhibited a negative correlation with daily influenza cases, while relative humidity and rainfall showed a positive correlation. Therefore, mean temperature, sunshine hours, relative humidity and rainfall were selected as research variables for further analysis. In addition, a significant negative correlation was found between mean temperature and atmospheric pressure (*r* = −0.88, *p* < 0.01). There was also a significant positive correlation between sunshine hours and daily temperature range (*r* = 0.78, *p* < 0.01). To avoid collinearity, atmospheric pressure and daily temperature range were removed from the models, and wind speed was included as a confounding variable to adjust models.

**Table 2:** Spearman’s correlation coefficients matrix of meteorological variables on influenza cases of children in Guangzhou, China, 2019–2022.

Variables	Influenza	Tmean	DTR	RH	AP	Rainfall	WH	SH
Influenza	1.00							
Tmean	−0.06^*^	1.00						
DTR	0.00	0.00	1.00					
RH	0.12^**^	0.15^**^	−0.51^**^	1.00				
AP	−0.01	**−0.88** ^**^	0.16^**^	−0.34^**^	1.00			
Rainfall	0.07^*^	0.13^**^	−0.50^**^	0.69^**^	−0.34^**^	1.00		
WS	−0.03	−0.21^**^	−0.18^**^	−0.35^**^	0.20^**^	−0.07^*^	1.00	
SH	−0.07^**^	0.26^**^	**0.78** ^**^	−0.59^**^	−0.05	−0.51^**^	−0.03	1.00

^*^p < 0.05; ^**^p < 0.01; Bold represented strong correlations. Influenza, all influenza cases; Tmean, mean temperature; DTR, daily temperature range; RH, mean relative humidity; AP, mean atmospheric pressure; Rainfall, aggregate rainfall; WS, mean wind speed; SH, sunshine hours.

### 3.3 Overall effects of meteorological variables on influenza incidence in children

**Figure 2:**
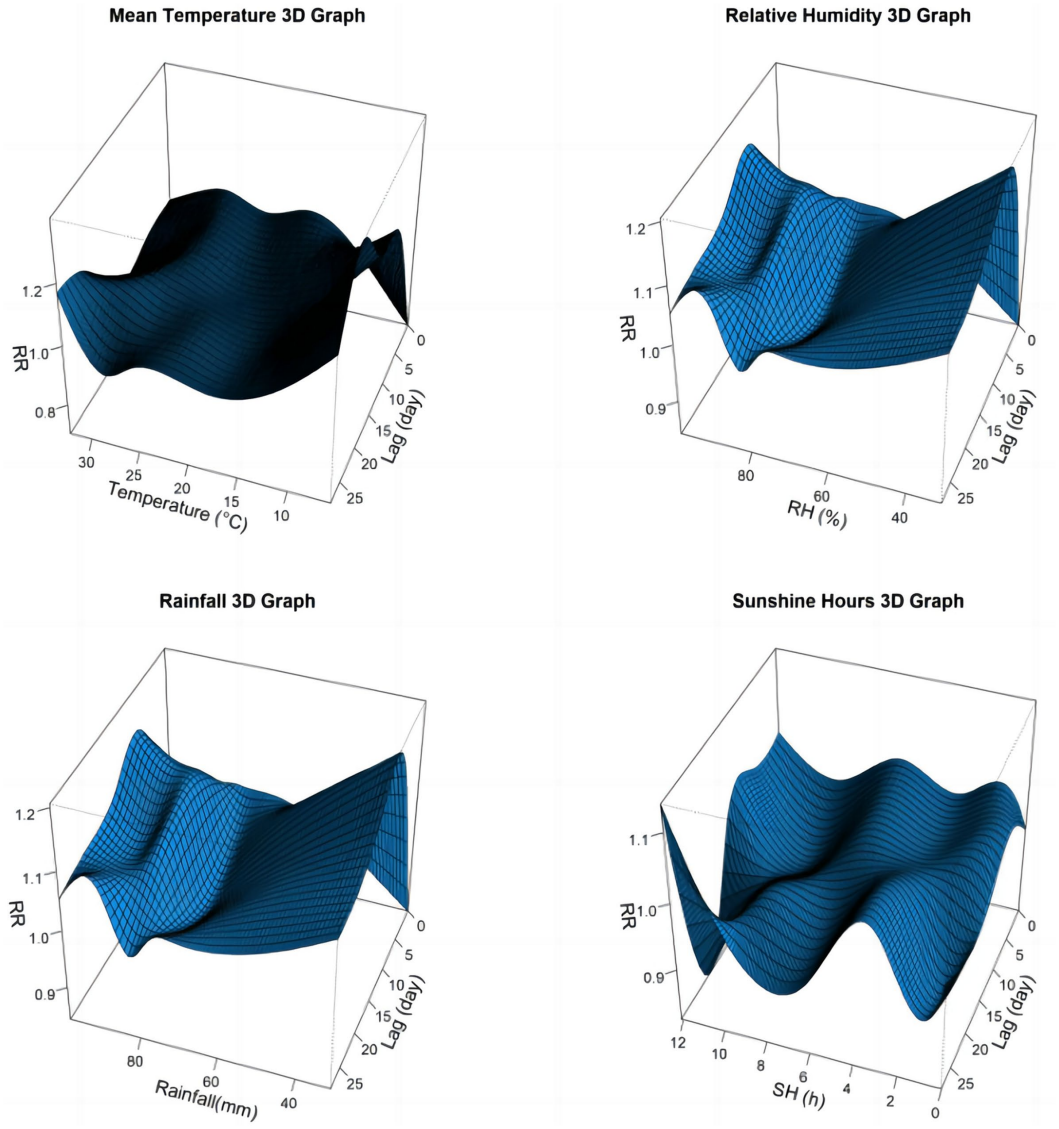
Three-dimensional graph of the relative risks of meteorological factors on children influenza cases in Guangzhou, China, 2019–2022.

**Figure 3:**
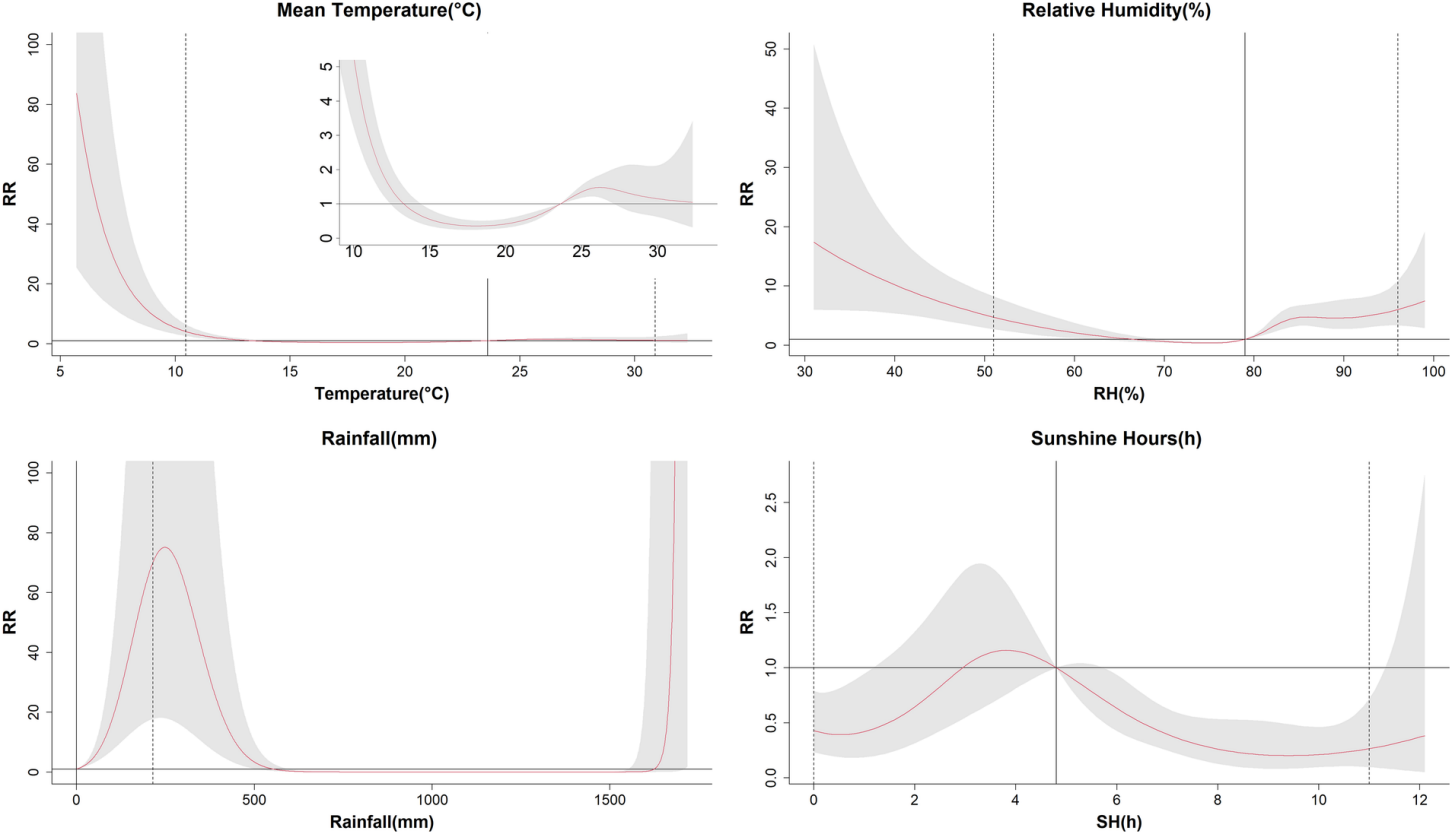
The estimated overall effects of mean temperature, relative humidity, aggregate rainfall, and sunshine on influenza cases of children in Guangzhou, China, 2019–2022. The median values of each meteorological variables (mean temperature: 23.6°C, relative humidity: 79%, rainfall: 0 mm, sunshine hours: 4.8 h/d) were as the reference levels. The Y lab represented the value of relative risk, the X lab represented the value of relevant variables. The red lines represented mean relative risks and gray regions were 95% confidence intervals. The black vertical line represented the medians of the meteorological factors, and the dotted lines represented the 2.5 percentile and the 97.5 percentile for the meteorological factors, respectively.

Figure 2 illustrated the relationships between meteorological variables and influenza incidence among children in Guangzhou with different lag days. For a better interpretation, one-dimensional curves were plotted to depict the overall effects of meteorological variables on influenza in Figure 3.

The relationship between average temperature and influenza cases generally presented a wavy pattern (Figure 3). When the temperature dropped below 13°C, RR increased sharply with decreasing temperature, peaking at 5.7°C with an RR of 83.78 (95% CI: 25.52, 275.09). Temperatures between 14°C and the median value showed a protective effect on influenza incidence, with the lowest RR recorded at 18.1°C (RR = 0.35, 95% CI: 0.24, 0.51). Above the reference value, higher temperatures posed a risk effect, but no statistically significant effect on influenza cases in children was observed when temperature exceeded 27°C (Table 3). In the temperature exposure-effect curves stratified by sex and age, the temperature-RR curve for males, females, 4–6 years group, 7–12 years group, and 13–17 years group were similar to the overall population curve. However the 7–12 years group had a substantially higher maximum RR (RR = 5501.18, 95% CI: 902.05, 33549.24) compared to other groups. The 0–3 years group showed some distinct characteristics, with no statistical significance observed at the highest and lowest temperature, but it exhibited a protective effect below the median and a risk effect above the median (Supplementary Figure S2).

**Table 3:** The overall effects of meteorological variables on influenza cases of children in Guangzhou, China, 2019–2022.

Variables	Low peak		High peak	
	Values	RR (95%CI)	Values	RR (95%CI)
T mean	18.1 (°C)	0.35 (0.24,0.51)^*^	5.7 (°C)	83.78 (25.52,275.09)^*^
RH	75 (%)	0.40 (0.27,0.57)^*^	31 (%)	17.40 (5.97,50.74)^*^
	/	/	99 (%)	7.50 (2.92,19.25)^*^
Rainfall	1,202 (mm)	0 (0,0)	248 (mm)	75.14 (18.01,313.51)^*^
	/	/	1,718 (mm)	2566.29 (1.85,3558574.07)^*^
SH	0.5 (h)	0.39 (0.19,0.81)^*^	3.8 (h)	1.16 (0.76,1.77)
	9.4 (h)	0.20 (0.08,0.49)^*^	/	/

^*^Overall effects of meteorological variables on influenza cases of children are significant at 95% confidence intervals; RR, relative risks; T mean, mean temperature; RH, mean relative humidity; Rainfall, aggregate rainfall; SH, sunshine hours.

The correlation between relative humidity and influenza cases showed a U-shape curve (Figure 3). The RR was increased when the relative humidity was below 66% or above 79%, with a median relative humidity of 79% used as the reference level. The highest RR was7.50 (95% CI: 2.92, 19.25) at 99%, while the lowest RR was 0.40 (95% CI: 0.27, 0.57) at 75%. In terms of gender and age stratification, the humidity-RR curves for males, females, and the 4–6 years group were similar to the overall population curve. For the 0–3 years group, lower relative humidity presented a protective effect, while higher relative humidity showed a risk effect. Both the 7–12 years and 13–17 years groups had the highest RRs when the relative humidity was the lowest (Supplementary Figure S3).

The association between aggregate rainfall and influenza cases showed a risk effect for rainfall below 550 mm and above 1,625 mm. The RR was increased exponentially when the rainfall exceeded 1,625 mm, reaching a maximum value of 2566.29 (95% CI: 1.85, 3558574.07) at the highest rainfall levels. Similar patterns were observed across different gender and age groups (Supplementary Figure S4).

The relationship between sunshine hours and influenza cases exhibited a fluctuating pattern, with both low and high sunshine duration were associated with reduced incidence of influenza. The lowest RR was 0.20 (95% CI: 0.08, 0.49) at 9.4 h. The SH-RR curve produced similar results when stratified by gender. In the 0–3 age group, sunlight duration of more than 5.8 h had a protective effect. On the contrary, the 7–12 age group had a protective effect only when sunlight duration was less than 2.3 h. The sunlight duration in the 13–17 age group did not show statistical significance throughout the study period (Supplementary Figure S5).

### 3.4 Extreme effects of meteorological variables on influenza incidence in children

**Figure 4:**
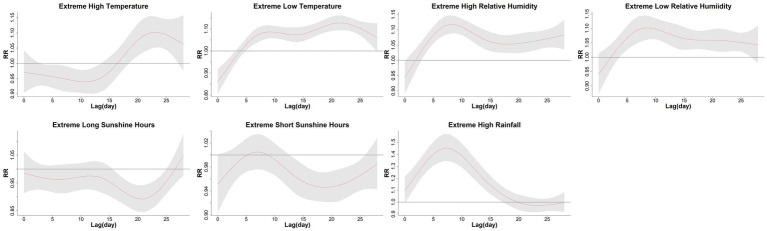
The extreme effects of mean temperature, relative humidity, sunshine hours, and rainfall on influenza cases of children along the lag days in Guangzhou, China, 2019–2022. The extreme high effect was calculated by comparing the 97.5th percentiles to median values, and the extreme low effect was calculated by comparing the 2.5th percentiles to median values. The median value of mean temperature, relative humidity, sunshine hours, and rainfall was 23.6°C, 79%, 4.8 h/d, and 0 mm, respectively. The Y lab represented the values of relative risks, the X lab represented the values of lag days. The red lines represented mean relative risks and gray regions were 95% confidence intervals.

The extreme effects of mean temperature, relative humidity, sunshine hours, and rainfall on influenza cases along the lag days were shown in Figure 4 and Table 4. High temperature demonstrated a protective effect during 3–13 lag days, but posed a risk effect within 18–27 lag days. The highest and lowest RRs for the hot effect were 1.10 (95% CI: 1.05, 1.15) and 0.94 (95% CI: 0.90, 0.98), respectively. However, low temperature exhibited a protective effect in the first 3 days and a risk effect after 5 days. The highest and lowest RRs for the cold effect were 1.13 (95% CI: 1.09, 1.16) and 0.86 (95% CI: 0.81, 0.92), respectively. The effects of both high and low humidity showed no statistical significance in the initial 3 days but became risk factors later on. The maximum RRs for the humid and dry effects were 1.12 (95% CI: 1.09, 1.15) and 1.10 (95% CI: 1.06, 1.14), respectively. Both long and short sunshine hours had protective effects within 15–25 lag days, while they were not statistically significant on other lag days. The minimum RRs for long sunshine hours and short sunshine hours were 0.89 (95% CI: 0.84, 0.94) and 0.95 (95% CI: 0.92, 0.97), respectively. With rainfall being absent on 59.1% of the days, we only estimated the extremely high effect of rainfall, which exhibited a risk effect with a lag of 1–17 days, and had a maximum RR of 1.45 (95% CI: 1.34,1.57).

**Table 4:** The extreme effects of meteorological variables on influenza cases of children along the lag days in Guangzhou, China, 2019–2022.

Variables	Lag days	Extreme high effect (97.5%)		Lag days	Extreme low effect (2.5%)	
		RR	95%CI		RR	95%CI
T mean	11	0.94^*^	0.90–0.98	0	0.86^*^	0.81–0.92
	23	1.10^*^	1.05–1.15	21	1.13^*^	1.09–1.16
SH	0	0.94^*^	0.89–0.99	0	0.94	0.87–1.01
	8	1.12^*^	1.09–1.15	9	1.10^*^	1.06–1.14
Rainfall	23	0.97	0.92–1.03	/	/	/
	7	1.45^*^	1.34–1.57	/	/	/
RH	21	0.89^*^	0.84–0.94	19	0.95^*^	0.92–0.97
	28	1.05	0.98–1.13	7	1.01	0.98–1.04

^*^Extreme effects of meteorological variables on influenza cases of children are significant at 95% confidence intervals. Since both the median and the 2.5th percentile of rainfall were 0, extreme low effect could not be calculated. RR, relative risks; T mean, mean temperature; RH, mean relative humidity; Rainfall, aggregate rainfall; SH, sunshine hours.

The cumulative extreme effects of meteorological factors on influenza were also calculated (Table 5). For children of all age, when comparing the 97.5^th^ percentiles to the median values, the cumulative RRs for the hot effect, humid effect, high rainfall effect, and long sunshine hours effect were 1.10 (95% CI: 0.52, 2.36), 6.04(95% CI: 3.36, 10.85), 70.21(95% CI: 17.75, 277.67) and 0.27(95% CI: 0.10, 0.72), respectively. When comparing the 2.5th percentiles to the median values, the cumulative RRs for the cold effect, dry effect, and short sunshine hours effect were 4.23 (95% CI: 2.67, 6.70), 4.76 (95% CI: 2.74, 8.26), and 0.43 (95% CI: 0.23, 0.79), respectively. Among the subpopulations, the hot effect showed statistical significance in all the subgroups, while the cold effect was not statistically significant in any subgroup. The effects of high rainfall and humidity showed statistical significance in all subgroups. Both long and short sunshine hours showed protective effects in all subgroups, although a few subgroups were not statistically significant. The 0–3 years age group presented protective effects for low temperature and drought, while other subgroups showed risk effects. The cumulative extreme effects showed minimal differences between males and females and were similar to those observed in the overall population.

**Table 5:** The cumulative extreme effects of meteorological variables on influenza cases of children by gender and age group.

Variables	Groups	Relative risks (95%)	
		Cumulative extreme low effect (2.5%)	Cumulative extreme high effect (97.5%)
T mean	Total	4.23(2.67,6.70)^*^	1.10(0.52,2.36)
	Male	4.38(2.71,7.06)^*^	1.28(0.59,2.78)
	Female	4.02(2.46,6.57)^*^	0.89(0.39,2.03)
	0–3 years	0.38(0.23,0.65)^*^	1.33(0.67,2.63)
	4–6 years	2.47(1.45,4.20)^*^	0.71(0.30,1.65)
	7–12 years	39.67(19.62,80.22)^*^	1.50(0.36,6.19)
	13–17 years	4.29(1.93,9.56)^*^	0.87(0.18,4.07)
RH	Total	4.76(2.74,8.26)^*^	6.04(3.36,10.85)^*^
	Male	4.74(2.66,8.45)^*^	5.73(3.11,10.57)^*^
	Female	4.80(2.68,8.59)^*^	6.44(3.46,11.98)^*^
	0–3 years	0.28(0.11,0.68)^*^	2.63(1.23,5.64)^*^
	4–6 years	1.63(0.80,3.31)	4.53(2.31,8.88)^*^
	7–12 years	23.32(12.49,43.57)^*^	11.18(4.95,25.26)^*^
	13–17 years	4.13(1.68,10.13)^*^	4.92(1.99,12.20)^*^
Rainfall	Total	/	70.21(17.75,277.67)^*^
	Male	/	71.64(17.64,290.91)^*^
	Female	/	67.58(15.76,289.77)^*^
	0–3 years	/	21.65(5.75,81.53)^*^
	4–6 years	/	55.10(13.29,228.54)^*^
	7–12 years	/	235.27(29.35,1886.12)^*^
	13–17 years	/	476.93(50.24,4527.93)^*^
SH	Total	0.43(0.23,0.79)^*^	0.27(0.10,0.72)^*^
	Male	0.42(0.23,0.78)^*^	0.30(0.11,0.81)^*^
	Female	0.44(0.23,0.85)^*^	0.23(0.08,0.66)^*^
	0–3 years	0.64(0.37,1.11)	0.28(0.13,0.61)^*^
	4–6 years	0.38(0.20,0.72)^*^	0.15(0.06,0.43)^*^
	7–12 years	0.22(0.07,0.68)^*^	0.14(0.02,1.29)
	13–17 years	0.29(0.09,0.93)^*^	0.26(0.03,2.05)

^*^The cumulative extreme effects of meteorological variables on influenza cases of children are significant at 95% confidence intervals. Since both the median and the 2.5th percentile of rainfall were 0, the cumulative extreme low effect could not be calculated. T mean, mean temperature; RH, mean relative humidity; Rainfall, aggregate rainfall; SH, sunshine hours.

## 4 Discussion

In this study, we observed two epidemic peaks of influenza cases among children in Guangzhou, which occurred during the winter of 2019–2020 and the summer of 2022. Average temperature, relative humidity, rainfall, and sunshine duration were found to be significant factors in influenza transmission. The correlation between relative humidity and influenza cases was illustrated by a U-shaped curve. The relationship between average temperature, rainfall, sunshine hours, and influenza cases presented a wavy pattern.

The incidence of influenza remained at an extremely low level from spring 2020 to the end of 2021, which might be related to the COVID-19 epidemic. COVID-19 was first identified in Wuhan, China, in December 2019, causing significant public health problems. Both SARS-CoV-2 and influenza viruses are respiratory viruses with many similarities (25). After the outbreak of COVID-19, various public health measures and non-pharmaceutical interventions were implemented, including frequent handwashing, mask-wearing, regular ventilation, and reduced gathering, which effectively prevented influenza transmission (26–28). As for the second influenza peak in 2022, this outbreak may be primarily attributed to the diminished immunity level and insufficient immune barrier in the population resulting from the low incidence of influenza over the previous 2 years, coupled with the normalization of COVID-19 prevention and control measures. Therefore, in the post-COVID era, influenza viruses have the potential to prevail once again as the predominant respiratory pathogen. China lifted a series of prevention and control measures against COVID-19 at the end of 2022, which weakened people’s initiative in preventing respiratory viruses, allowing influenza viruses to take advantage. It is essential for governments and medical institutions to enhance surveillance of influenza symptoms and continue promoting non-pharmaceutical interventions as preventive measures against influenza.

Our study found that low temperatures can dramatically increase influenza activity, which is consistent with the results in many temperate and subtropical regions (9–11, 24, 29). Animal experiments have shown that influenza viruses are more stable and have a longer half-life, and influenza virus titers in guinea pigs are higher at low temperatures (30, 31), which demonstrates that influenza viruses have a stronger ability to transmit under low-temperature conditions. Furthermore, low temperature can affect the immune system function and increase the susceptibility of the population (32), thus promoting the occurrence of influenza. At low temperatures, mucus secretion in the nasal mucosa increases and the frequency of ciliary movement decreases (33). Cold air slows down the clearance of mucociliary, thereby promoting virus transmission in the respiratory tract. Moreover, temperature can affect the spread of influenza by altering human activity. As the temperature drops, people tend to rely more on heating systems, minimize ventilation, and spend more time indoors (34, 35). Indoor crowding increases the opportunities and duration of person-to-person contact, thereby facilitating the transmission of influenza.

Consistent with other studies (9, 10, 29, 36–38), our studies confirmed a U-shaped relationship between relative humidity and influenza incidence. The risk of influenza increased with low (<66%) or high (>79%) relative humidity, and the detrimental effects of both arid and humid weather lasted for a duration of 26 days. When the relative humidity is low, droplets containing the influenza virus rapidly dehydrate and form droplet nuclei upon expulsion, thereby prolonging the survival time of virus and maintaining its stability. In addition, droplet nuclei can remain suspended in the environment for extended periods, increasing the opportunity for people inhaling influenza virus. Low humidity can also cause dryness in the nose, throat, and respiratory tract, lowing immunity and making individuals more susceptible to virus attacks (31, 39–41). Moreover, the droplets expelled by an infected source combine with the moisture in the air under high relative humidity conditions, increasing the volume and weight of the droplets, which accelerates the rate of sedimentation and exposes the population to a higher risk of influenza virus transmission (31, 42).

There is a lack of consensus regarding the relationship between rainfall and influenza incidence. Our research found that compared to the absence of precipitation, both precipitation levels below 550 mm and above 1,625 mm were associated with increased risk of influenza, while a precipitation range of 550–1,625 mm showed a protective effect. One possible explanation for this result is the limited number of rainy days and the wide range of rainfall (0–1,718 mm), which may impact the statistical precision and accuracy. This is one of the reasons for the different results compared to the previous study in Guangzhou (11). Tamerius et al. have shown that influenza activity was more likely to peak during rainy months (35). Similarly, two studies conducted in Hong Kong and Hangzhou, China, demonstrated a significant positive correlation between rainfall and influenza activity (43, 44). In contrast, Murray et al. found an inverse correlation between influenza activity and precipitation in Egypt (45). Besides, some studies did not find an association between rainfall and influenza (11, 46). In general, the impact of rainfall on influenza varies by region (47), suggesting that the effect of climate on influenza is the result of the interaction of multiple meteorological factors. The mechanism through which rainfall affects influenza is still unclear, but commonly proposed explanations are included: (1) High rainfall leads to reduced outdoor activities and increased indoor activities, resulting in poor air circulation and increased contact and exposure time (48). (2) Rainfall is usually accompanied by a decrease in solar radiation, which can lower body immunity by affecting vitamin D synthesis (49). (3) Moderate rainfall can play an important role in cleaning the air and reducing the concentration of influenza virus particles in the atmosphere (47).

There is limited literature available on the relationship between sunshine hours and influenza. Our study illustrated that a higher sunshine duration reduced the risk of influenza activity, which was consistent with a previous study conducted in the subtropical region (10). Soebiyanto et al. showed that increasing solar radiation by 16.4 W/m (2) could lead to a 4.5–27.2% decrease in influenza (47). As mentioned above, sunshine can enhance the synthesis of vitamin D and strengthen immunity (49). It has also been suggested by Sagripanti et al. that solar radiation can inactivate viruses in the environment (50). However, we also observed that even low sunshine duration presented a protective effect. The specific reasons and mechanisms behind this require further exploration. In addition, the lag effects of long and short sunshine hours on influenza only became statistically significant after a two-weeks lag, indicating that the impact of sunshine duration on influenza is chronic, delayed, and not immediate. Based on our findings, we recommend that parents encourage their children to engage in more outdoor activities, and moderate sun exposure each day can help reduce the transmission of influenza virus.

Many studies have shown that multiple climatic factors can jointly affect the survival and transmission ability of the influenza virus (35, 51, 52). The risk of seasonal influenza significantly rises in “cold-dry,” “hot-humid” and “humid-rainy” conditions (35, 52). Considering the substantial disparities in temperature, humidity, and rainfall between subtropical and temperate regions, this partly explains why subtropical areas like Guangzhou experience two epidemic peaks in wither and summer, whereas temperate regions have only one epidemic peak each year. As a consequence, our study suggested that reducing the incidence of influenza can be achieved by raising the temperature and humidity in winter, as well as implementing cooling and dehumidification measures in summer.

The relationship between meteorological factors and influenza was further explored by stratifying gender and age. No significant difference was observed between males and females. The impacts of cumulative extreme low temperature and low relative humidity on influenza among children aged 0–3 presented protective effects, while they showed risk effects in other age groups. Moreover, the 0–3 years group had the lowest RRs of cumulative extreme high relative humidity and rainfall, suggesting that the risk effect among children aged 0–3 was smaller than that of other age groups. Despite some slight variations in age categorization, this particular finding was not observed in a similar study conducted in Guangzhou in 2019 (11). This phenomenon can be explained by the fact that preschoolers (0–3 years old) have fewer social channels and engage in fewer clustered activities. Extreme weather conditions further limit the range and duration of activity for preschoolers, thereby reducing their exposure to the influenza virus. In addition, infants aged 0–6 months carry maternal immune antibodies that effectively resist the invasion of the influenza virus. However, these maternal immune antibodies gradually disappear after 12 months, and the autoimmune barrier has not yet been well established, leaving infants susceptible to the influenza virus during this period. Meanwhile, the highest RRs of cumulative extreme effect of all meteorological factors (except sunshine hours) were observed in the 7–12 years group. Compared to middle school students (13–17 years old) who resided in boarding schools, most primary school students (7–12 years old) have to commute between school and home every day, increasing the likelihood of transmitting the influenza virus to classmates, adult family members, and other community members. Many studies have demonstrated that school closures during influenza outbreaks not only reduce influenza transmission within the school but also in the community (53, 54). Moreover, young children are more prone to poor hand hygiene, such as touching contaminated objects and then touching their nose, eyes, and mouth (55). These bad behaviors expedite the velocity and range of influenza transmission. As a consequence, greater attention should be given to the prevention of influenza among primary school students.

All in all, meteorological factors affect influenza transmission in three major ways: (1) By affecting virus viability and infectivity. (2) By influencing population contact opportunities. (3) By impacting the immunity of susceptible individuals. Influenza vaccination is the most effective approach to prevent influenza. The WHO recommends annual seasonal influenza vaccination for children aged 6 to 59 months. However, the vaccination rate for the entire population in China is only about 2% (56). Promoting the influenza vaccine and increasing the vaccination rate remain fundamental and crucial tasks in preventing influenza. Our research suggests that cold-dry and wet-rainy conditions can facilitate the spread of influenza, and the maximum benefits of influenza vaccination can be obtained during these periods. Taking into account the climate conditions in Guangzhou and considering the immune response time, we recommend promoting the vaccination program in Guangzhou from April to May and October to November each year. Furthermore, our research findings also indicate that meteorological factors have the greatest impact on children aged 7–12. Therefore, priority should be given to vaccinating primary school students against influenza during the aforementioned periods.

This study has several limitations. First, some potentially influential factors have not been controlled, such as host susceptibility, vaccination, socioeconomic level, air pollutants, and health policies. Particularly, we have to consider the impact of the COVID-19 epidemic on influenza, which was the primary reason why the results differed from previous studies. Second, we only explored the effect of a single meteorological factor on influenza. Further research is needed to explore the interaction between meteorological factors and their combined impact on influenza. Third, meteorological data were measured outdoors, while children spend most of their time indoors. This discrepancy in temperature and humidity between indoor and outdoor environments may have influenced the results. Fourth, only the data from Guangzhou, China, were analyzed, so the conclusions cannot stand for other cities and are difficult to extrapolate. Finally, due to the short study period, we were unable to reveal the long-term trends of influenza in the post-COVID-19 era.

However, the ongoing COVID-19 pandemic has shifted attention away from influenza in recent years. Our study provides a timely contribution to the existing research on the relationship between meteorological factors and influenza during the COVID-19 epidemic. Compared to the previous study conducted in Guangzhou, our research complemented the correlations between precipitation and sunshine duration with influenza, providing valuable references for the future development of influenza prevention and control policies in Guangzhou. Furthermore, our results can offer scientific guidance for predicting the peak of the influenza epidemic, determining the optimal timing of vaccination, and developing non-pharmaceutical intervention strategies for influenza.

## 5 Conclusion

We identified a significant nonlinear correlation between multiple meteorological factors and the incidence influenza incidence among children in Guangzhou, with lag effects taken into account. Accordingly, temperature, relative humidity, rainfall, and sunshine hours can be used as important predictors of influenza in children to improve the early warning system of influenza. Low temperatures, both high and low relative humidity, and high rainfall contribute to the transmission of influenza. Both long and short sunshine hours can reduce the incidence of influenza. Extreme weather reduces the risk of influenza in the age group of 0–3 years, but significantly increases the risk for those aged 7–12 years. Influenza vaccine was encouraged to promoted in April–May and October–November in Guangzhou, with primary school students receiving priority access to the influenza vaccine during these periods.

## Data availability statement

The original contributions presented in the study are included in the article/Supplementary material, further inquiries can be directed to the corresponding authors.

## Ethics statement

Ethical approval was not required for the study involving humans in accordance with the local legislation and institutional requirements. Written informed consent to participate in this study was not required from the participants or the participants’ legal guardians/next of kin in accordance with the national legislation and the institutional requirements.

## Author contributions

ZC: Methodology, Investigation, Validation, Visualization, Writing – original draft. YL: Conceptualization, Data curation, Writing – review & editing. HY: Data curation, Writing – review & editing. JC: Conceptualization, Supervision, Writing – review & editing. XH: Software, Writing – original draft. LZ: Supervision, Writing – review & editing. BL: Methodology, Writing – review & editing. GL: Methodology, Supervision, Writing – review & editing. PQ: Conceptualization, Resources, Writing – review & editing. WF: Data curation, Resources, Writing – review & editing. DWa: Conceptualization, Methodology, Resources, Supervision, Writing – review & editing. DWu: Conceptualization, Funding acquisition, Methodology, Supervision, Writing – review & editing.

## Footnotes

^1^ http://gd.cma.gov.cn/

## References

[1] Nypaver C Dehlinger C Carter C. Influenza and Influenza Vaccine: A Review. J. Midwifery Womens Health. (2021) 66:45–53. doi: 10.1111/jmwh.13203, PMID: 33522695 PMC8014756

[2] World Health Organization. Influenza (Seasonal) (2023). Available at: https://www.who.int/news-room/fact-sheets/detail/influenza-(seasonal)

[3] Krammer F Smith GJD Fouchier RAM Peiris M Kedzierska K Doherty PC. Influenza. Nat. Rev. Dis. Primers. (2018) 4:3. doi: 10.1038/s41572-018-0002-y, PMID: 29955068 PMC7097467

[4] He Z Tao H. Epidemiology and ARIMA model of positive-rate of influenza viruses among children in Wuhan, China: a nine-year retrospective study. Int. J. Infect. Dis. (2018) 74:61–70. doi: 10.1016/j.ijid.2018.07.003, PMID: 29990540

[5] Nair H Brooks WA Katz M Roca A Berkley JA Madhi SA. Global burden of respiratory infections due to seasonal influenza in young children: a systematic review and meta-analysis. Lancet. (2011) 378:1917–30. doi: 10.1016/S0140-6736(11)61051-9 22078723

[6] Iuliano AD Roguski KM Chang HH Muscatello DJ Palekar R Tempia S. Estimates of global seasonal influenza-associated respiratory mortality: a modelling study. Lancet. (2018) 391:1285–300. doi: 10.1016/S0140-6736(17)33293-2, PMID: 29248255 PMC5935243

[7] Poehling KA Edwards KM Weinberg GA Szilagyi P Staat MA Iwane MK. The underrecognized burden of influenza in young children. N. Engl. J. Med. (2006) 355:31–40. doi: 10.1056/NEJMoa054869, PMID: 16822994

[8] Lafond KE Nair H Rasooly MH Valente F Booy R Rahman M. Global role and burden of influenza in pediatric respiratory hospitalizations, 1982-2012: a systematic analysis. PLoS Med. (2016) 13:e1001977. doi: 10.1371/journal.pmed.1001977, PMID: 27011229 PMC4807087

[9] Park JE Son WS Ryu Y Choi SB Kwon O Ahn I. Effects of temperature, humidity, and diurnal temperature range on influenza incidence in a temperate region. Influenza Other Respi. Viruses. (2020) 14:11–8. doi: 10.1111/irv.12682, PMID: 31631558 PMC6928031

[10] Qi L Liu T Gao Y Tian D Tang W Li Q. Effect of meteorological factors on the activity of influenza in Chongqing, China, 2012-2019. PloS One. (2021) 16:e0246023. doi: 10.1371/journal.pone.0246023, PMID: 33534840 PMC7857549

[11] Guo Q Dong Z Zeng W Ma W Zhao D Sun X. The effects of meteorological factors on influenza among children in Guangzhou, China. Influenza Other Respi. Viruses. (2019) 13:166–75. doi: 10.1111/irv.12617, PMID: 30407738 PMC6379639

[12] Soebiyanto RP Clara W Jara J Castillo L Sorto OR Marinero S. The role of temperature and humidity on seasonal influenza in tropical areas: Guatemala, El Salvador and Panama, 2008-2013. PloS One. (2014) 9:e100659. doi: 10.1371/journal.pone.0100659, PMID: 24956184 PMC4067338

[13] Shimmei K Nakamura T Ng CFS Hashizume M Murakami Y Maruyama A. Association between seasonal influenza and absolute humidity: time-series analysis with daily surveillance data in Japan. Sci. Rep. (2020) 10:7764. doi: 10.1038/s41598-020-63712-2, PMID: 32385282 PMC7211015

[14] Chen C Zhang X Jiang D Yan D Guan Z Zhou Y. Associations between temperature and influenza activity: a National Time Series Study in China. Int. J. Environ. Res. Public Health. (2021) 18:846. doi: 10.3390/ijerph182010846 34682590 PMC8535740

[15] Viboud C Boelle PY Pakdaman K Carrat F Valleron AJ Flahault A. Influenza epidemics in the United States, France, and Australia, 1972-1997. Emerg. Infect. Dis. (2004) 10:32–9. doi: 10.3201/eid1001.020705 15078594 PMC3322745

[16] Finkelman BS Viboud C Koelle K Ferrari MJ Bharti N Grenfell BT. Global patterns in seasonal activity of influenza a/H3N2, a/H1N1, and B from 1997 to 2005: viral coexistence and latitudinal gradients. PloS One. (2007) 2:e1296. doi: 10.1371/journal.pone.0001296, PMID: 18074020 PMC2117904

[17] Viboud C Alonso WJ Simonsen L. Influenza in tropical regions. PLoS Med. (2006) 3:e89. doi: 10.1371/journal.pmed.0030089, PMID: 16509764 PMC1391975

[18] Moura FE Ac P Siqueira MM. Seasonality of influenza in the tropics: a distinct pattern in northeastern Brazil. Am. J. Trop. Med. Hyg. (2009) 81:180–3. doi: 10.4269/ajtmh.2009.81.180, PMID: 19556586

[19] Shi XZ Song ZJ Yang YL Bao JZ Zhu HL Luo ZX. Association and prediction of influenza-like illness with meteorological factors in Mississippi, USA. Biomed. Environ. Sci. (2022) 35:962–7. doi: 10.3967/bes2022.123, PMID: 36443274

[20] Gasparrini A. Distributed Lag Linear and Non-Linear Models in R: The Package dlnm. J. Stat. Softw. (2011) 43:1–20. doi: 10.18637/jss.v043.i08 PMC3191524 22003319

[21] Lu JY Chen ZQ Liu YH Liu WH Ma Y Li TG. Effect of meteorological factors on scarlet fever incidence in Guangzhou City, southern China, 2006-2017. Sci. Total Environ. (2019) 663:227–35. doi: 10.1016/j.scitotenv.2019.01.318, PMID: 30711589

[22] Lu J Yang Z Ma X Ma M Zhang Z. The role of meteorological factors on mumps incidence among children in Guangzhou, southern China. PloS One. (2020) 15:e0232273. doi: 10.1371/journal.pone.0232273, PMID: 32348370 PMC7190132

[23] Lei C Lou CT Io K SiTou KI Ip CP U HJ. Viral etiology among children hospitalized for acute respiratory tract infections and its association with meteorological factors and air pollutants: a time-series study (2014-2017) in Macao. BMC Infect. Dis. (2022) 22:588. doi: 10.1186/s12879-022-07585-y, PMID: 35786346 PMC9250746

[24] Yin J Liu T Tang F Chen D Sun L Song S. Effects of ambient temperature on influenza-like illness: A multicity analysis in Shandong Province, China, 2014-2017. Front. Public Health. (2023) 10:2296–565. doi: 10.3389/fpubh.2022.1095436 PMC9868675 36699880

[25] Chotpitayasunondh T Fischer TK Heraud JM Hurt AC Monto AS Osterhaus A. Influenza and COVID-19: what does co-existence mean? Influenza Other Respi. Viruses. (2021) 15:407–12. doi: 10.1111/irv.12824, PMID: 33128444 PMC8051702

[26] Chiu NC Chi H Tai YL Peng CC Tseng CY Chen CC. Impact of wearing masks, hand hygiene, and social distancing on influenza, enterovirus, and all-cause pneumonia during the coronavirus pandemic: retrospective National Epidemiological Surveillance Study. J. Med. Internet Res. (2020) 22:e21257. doi: 10.2196/21257, PMID: 32750008 PMC7471891

[27] Huang QS Wood T Jelley L Jennings T Jefferies S Daniells K. Impact of the COVID-19 nonpharmaceutical interventions on influenza and other respiratory viral infections in New Zealand. Nat. Commun. (2021) 12:1001. doi: 10.1038/s41467-021-21157-9, PMID: 33579926 PMC7881137

[28] Tran LK Huang DW Li NK Li LM Palacios JA Chang HH. The impact of the COVID-19 preventive measures on influenza transmission: molecular and epidemiological evidence. Int. J. Infect. Dis. (2022) 116:11–3. doi: 10.1016/j.ijid.2021.12.323, PMID: 34902583 PMC8662913

[29] Zhu H Chen S Lu W Chen K Feng Y Xie Z. Study on the influence of meteorological factors on influenza in different regions and predictions based on an LSTM algorithm. BMC Public Health. (2022) 22:2335. doi: 10.1186/s12889-022-14299-y, PMID: 36514013 PMC9745690

[30] Pica N Chou YY Bouvier NM Palese P. Transmission of influenza B viruses in the guinea pig. J. Virol. (2012) 86:4279–87. doi: 10.1128/JVI.06645-11, PMID: 22301149 PMC3318622

[31] Lowen AC Mubareka S Steel J Palese P. Influenza virus transmission is dependent on relative humidity and temperature. PLoS Pathog. (2007) 3:1470–6. doi: 10.1371/journal.ppat.0030151, PMID: 17953482 PMC2034399

[32] Ianevski A Zusinaite E Shtaida N Kallio-Kokko H Valkonen M Kantele A. Low temperature and low UV indexes correlated with peaks of influenza virus activity in northern Europe during 2010(−)2018. Viruses. (2019) 11:207. doi: 10.3390/v11030207, PMID: 30832226 PMC6466003

[33] Eccles R. An explanation for the seasonality of acute upper respiratory tract viral infections. Acta Otolaryngol. (2002) 122:183–91. doi: 10.1080/00016480252814207, PMID: 11936911

[34] Mikolajczyk RT Akmatov MK Rastin S Kretzschmar M. Social contacts of school children and the transmission of respiratory-spread pathogens. Epidemiol. Infect. (2008) 136:813–22. doi: 10.1017/S0950268807009181, PMID: 17634160 PMC2870867

[35] Tamerius JD Shaman J Alonso WJ Bloom-Feshbach K Uejio CK Comrie A. Environmental predictors of seasonal influenza epidemics across temperate and tropical climates. PLoS Pathog. (2013) 9:e1003194. doi: 10.1371/journal.ppat.1003194, PMID: 23505366 PMC3591336

[36] Bai YL Huang DS Liu J Li DQ Guan P. Effect of meteorological factors on influenza-like illness from 2012 to 2015 in Huludao, a northeastern city in China. PeerJ. (2019) 7:e6919. doi: 10.7717/peerj.6919, PMID: 31110929 PMC6501768

[37] Ng H Li Y Zhang T Lu Y Wong CH Ni J. Association between multiple meteorological variables and seasonal influenza a and B virus transmission in Macau. Heliyon. (2022) 8:e11820. doi: 10.1016/j.heliyon.2022.e11820, PMID: 36468127 PMC9713336

[38] Ali ST Cowling BJ Wong JY Chen D Shan S Lau EHY. Influenza seasonality and its environmental driving factors in mainland China and Hong Kong. Sci. Total Environ. (2022) 818:151724. doi: 10.1016/j.scitotenv.2021.151724 34800462

[39] Yang W Elankumaran S Marr LC. Relationship between humidity and influenza a viability in droplets and implications for influenza's seasonality. PloS One. (2012) 7:e46789. doi: 10.1371/journal.pone.0046789, PMID: 23056454 PMC3463543

[40] Shaman J Kohn M. Absolute humidity modulates influenza survival, transmission, and seasonality. Proc. Natl. Acad. Sci. U. S. A. (2009) 106:3243–8. doi: 10.1073/pnas.0806852106 19204283 PMC2651255

[41] Schaffer FL Soergel ME Straube DC. Survival of airborne influenza virus: effects of propagating host, relative humidity, and composition of spray fluids. Arch. Virol. (1976) 51:263–73. doi: 10.1007/BF01317930, PMID: 987765

[42] Noti JD Blachere FM McMillen CM Lindsley WG Kashon ML Slaughter DR. High humidity leads to loss of infectious influenza virus from simulated coughs. PloS One. (2013) 8:e57485. doi: 10.1371/journal.pone.0057485, PMID: 23460865 PMC3583861

[43] Lau SY Cheng W Yu Z Mohammad KN Wang MH Zee BC. Independent association between meteorological factors, PM2.5, and seasonal influenza activity in Hangzhou, Zhejiang province, China. Influenza Other Respi. Viruses. (2021) 15:513–20. doi: 10.1111/irv.12829, PMID: 33342077 PMC8189232

[44] Chong KC Goggins W Zee BC Wang MH. Identifying meteorological drivers for the seasonal variations of influenza infections in a subtropical city—Hong Kong. Int. J. Environ. Res. Public Health. (2015) 12:1560–76. doi: 10.3390/ijerph120201560, PMID: 25635916 PMC4344680

[45] Murray EJ Morse SS. Seasonal oscillation of human infection with influenza a/H5N1 in Egypt and Indonesia. PloS One. (2011) 6:e24042. doi: 10.1371/journal.pone.0024042, PMID: 21909409 PMC3164700

[46] Ma P Tang X Zhang L Wang X Wang W Zhang X. Influenza a and B outbreaks differed in their associations with climate conditions in Shenzhen, China. Int. J. Biometeorol. (2022) 66:163–73. doi: 10.1007/s00484-021-02204-y, PMID: 34693474 PMC8542503

[47] Soebiyanto RP Gross D Jorgensen P Buda S Bromberg M Kaufman Z. Associations between meteorological parameters and influenza activity in Berlin (Germany), Ljubljana (Slovenia), castile and Leon (Spain) and Israeli districts. PloS One. (2015) 10:e0134701. doi: 10.1371/journal.pone.0134701, PMID: 26309214 PMC4550247

[48] du Prel JB Puppe W Grondahl B Knuf M Weigl JA Schaaff F. Are meteorological parameters associated with acute respiratory tract infections? Clin. Infect. Dis. (2009) 49:861–8. doi: 10.1086/605435 19663691

[49] Cannell JJ Vieth R Umhau JC Holick MF Grant WB Madronich S. Epidemic influenza and vitamin D. Epidemiol. Infect. (2006) 134:1129–40. doi: 10.1017/S0950268806007175, PMID: 16959053 PMC2870528

[50] Sagripanti JL Lytle CD. Inactivation of influenza virus by solar radiation. Photochem. Photobiol. (2007) 83:1278–82. doi: 10.1111/j.1751-1097.2007.00177.x, PMID: 17880524

[51] Zhou L Yang H Pan W Xu J Feng Y Zhang W. Association between meteorological factors and the epidemics of influenza (sub)types in a subtropical basin of Southwest China. Epidemics. (2022) 41:100650. doi: 10.1016/j.epidem.2022.100650 36375312

[52] Wang XL Yang L He DH Chiu APY Chan KH Chan KP. Different responses of influenza epidemic to weather factors among Shanghai, Hong Kong, and British Columbia. Int. J. Biometeorol. (2017) 61:1043–53. doi: 10.1007/s00484-016-1284-y 28180957

[53] Jackson C Vynnycky E Hawker J Olowokure B Mangtani P. School closures and influenza: systematic review of epidemiological studies. BMJ Open. (2013) 3:e002149. doi: 10.1136/bmjopen-2012-002149, PMID: 23447463 PMC3586057

[54] Ali ST Cowling BJ Lau EHY Fang VJ Leung GM. Mitigation of influenza B epidemic with school closures, Hong Kong, 2018. Emerg. Infect. Dis. (2018) 24:2071–3. doi: 10.3201/eid2411.180612, PMID: 30334723 PMC6200008

[55] Nayak J Hoy G Gordon A. Influenza in Children. Cold Spring Harb. Perspect. Med. (2021) 11:8430. doi: 10.1101/cshperspect.a038430 PMC7778215 31871228

[56] Yang J Atkins KE Feng L Pang M Zheng Y Liu X. Seasonal influenza vaccination in China: landscape of diverse regional reimbursement policy, and budget impact analysis. Vaccine. (2016) 34:5724–35. doi: 10.1016/j.vaccine.2016.10.013 27745951

