# Supplementary material for: The role of meteorological factors on influenza incidence among children in Guangzhou China, 2019–2022
Source: Front Public Health. 2024 Jan 8;11:1268073. doi: 10.3389/fpubh.2023.1268073 (PMC10800649; doi:10.3389/fpubh.2023.1268073)
Supplement: Supplementary file 1 [file Data_Sheet_1.docx]

**Figure S1. scatter plot between meteorological factors and the daily number of influenza cases**


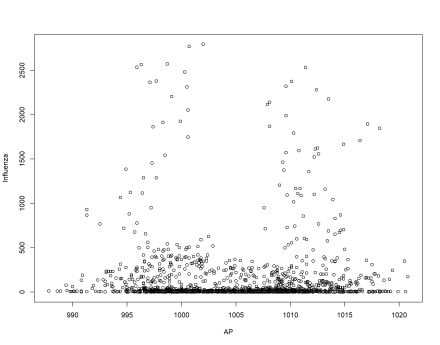

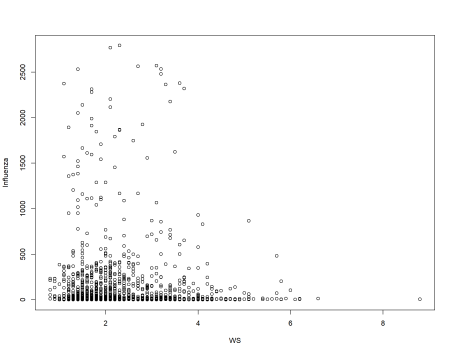


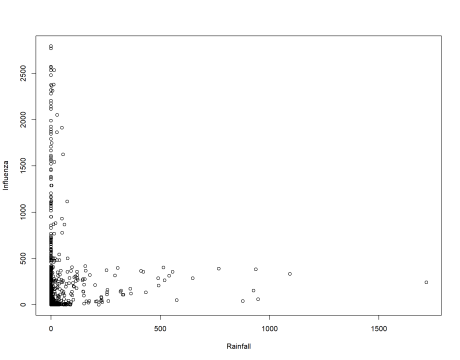

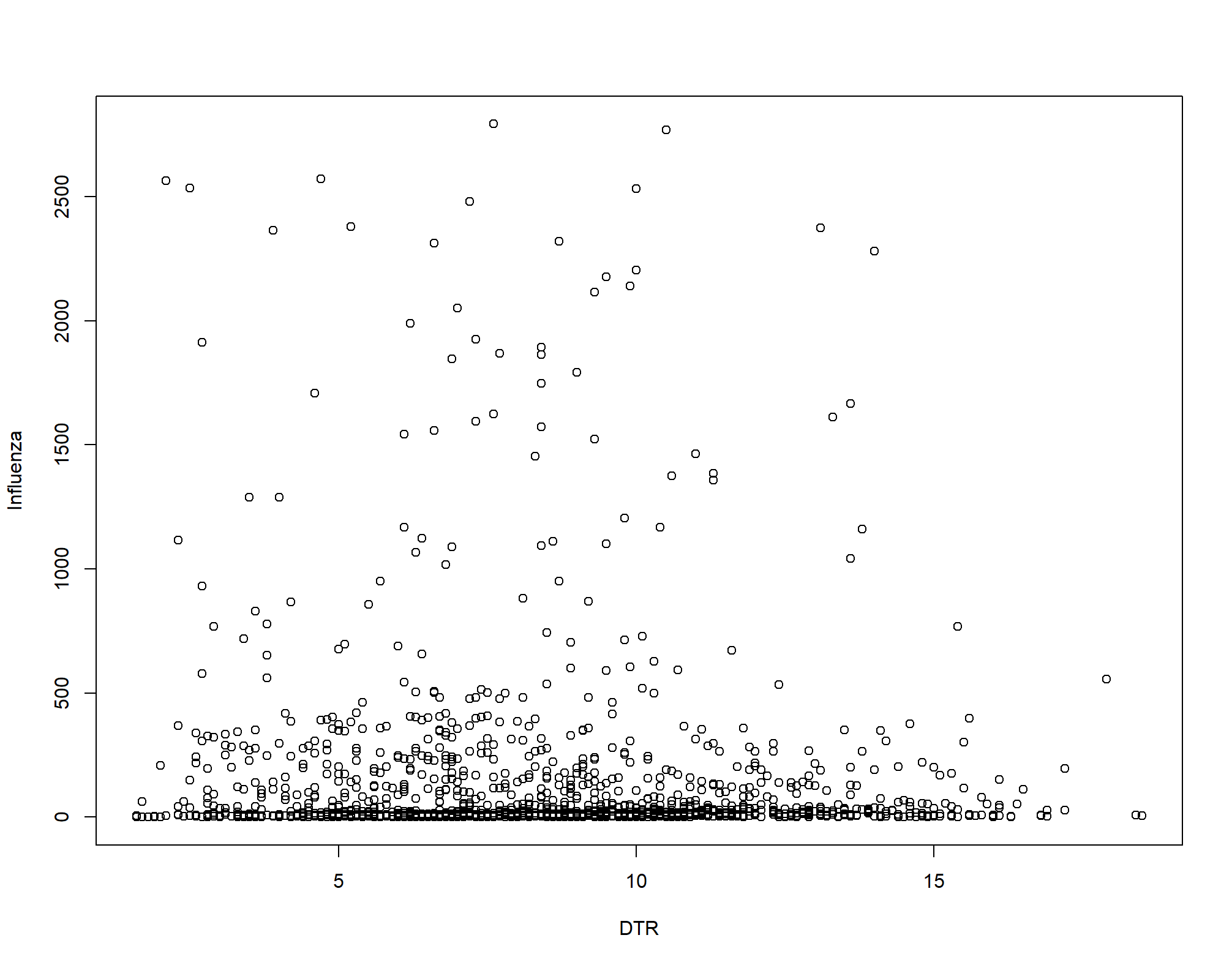


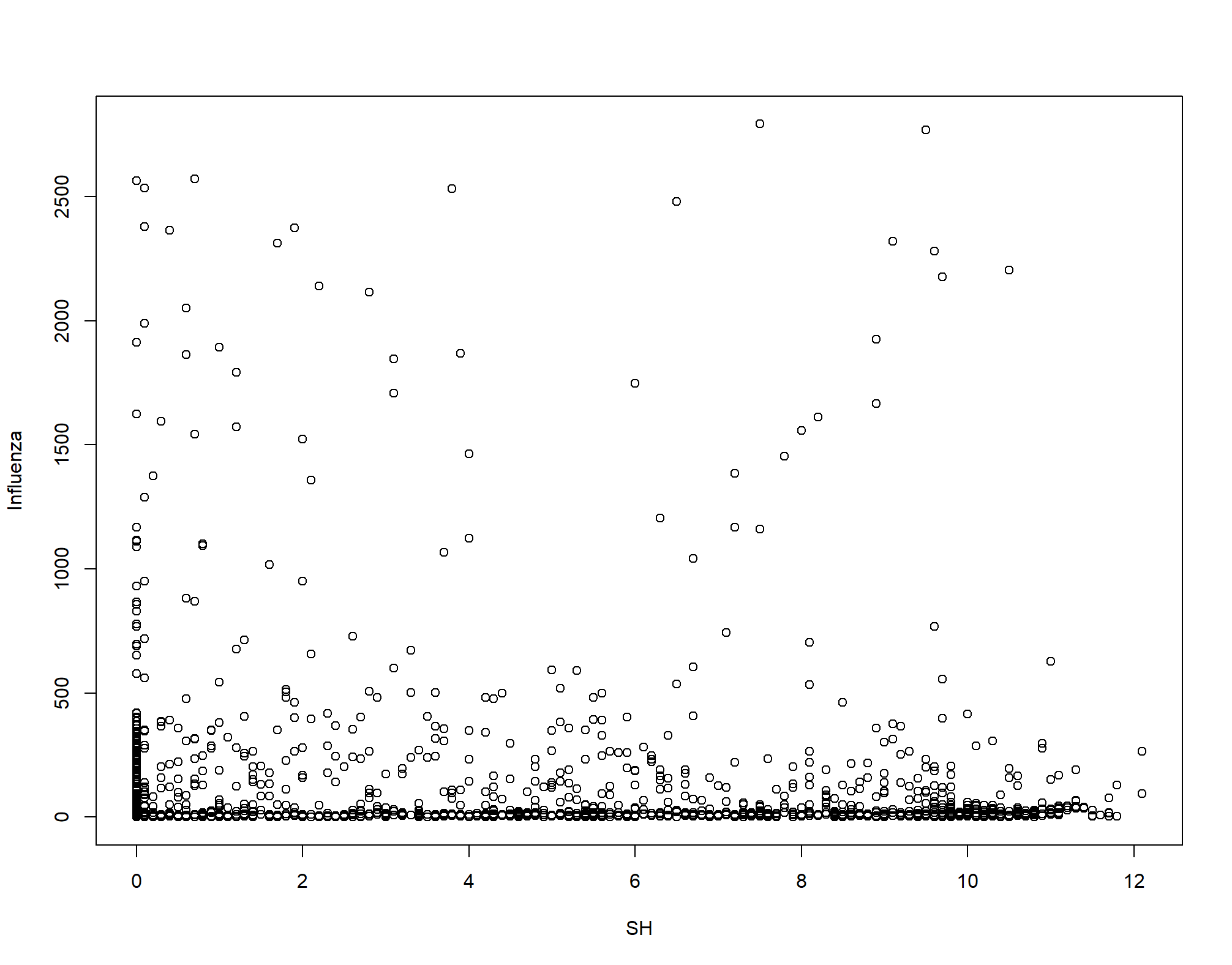

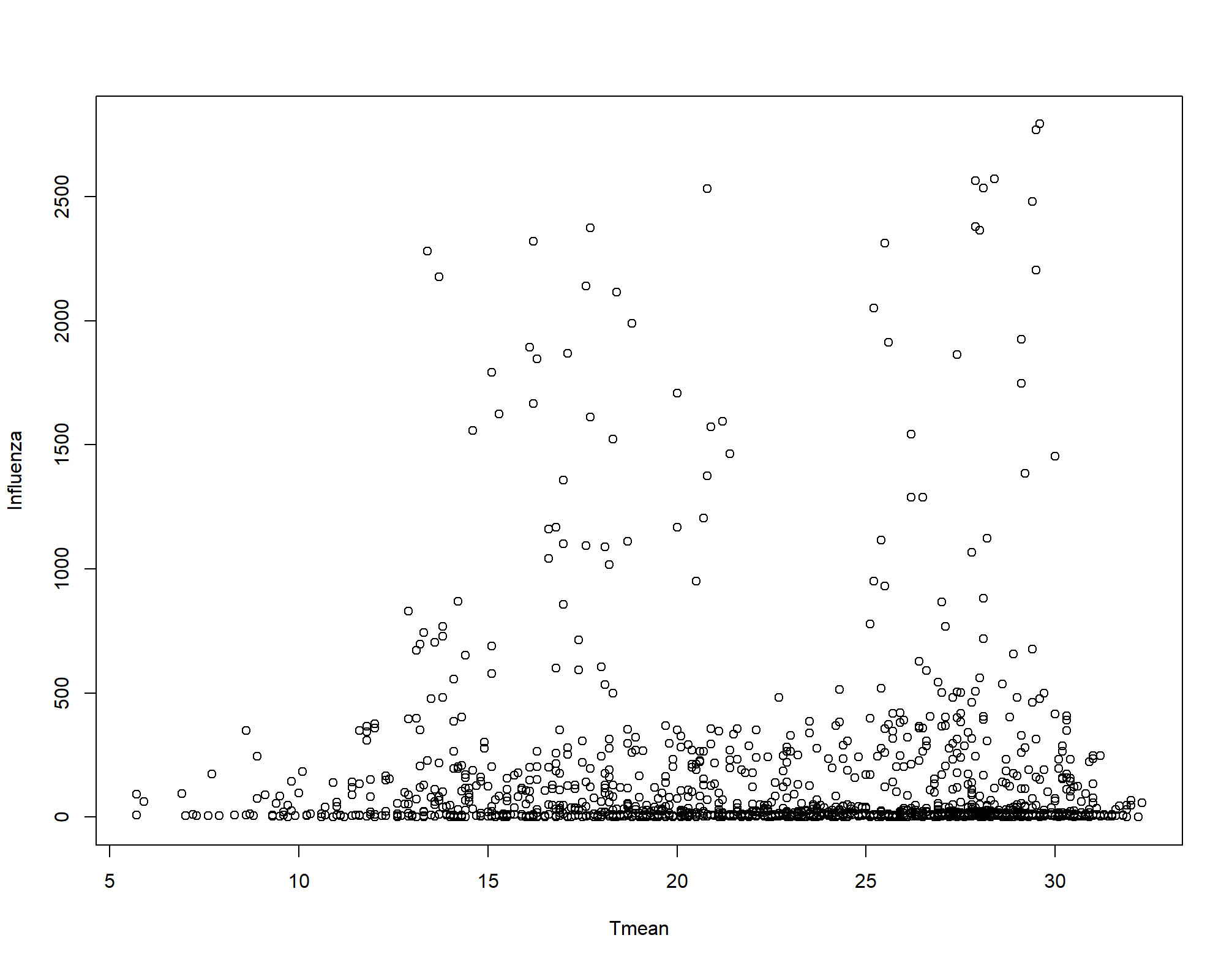


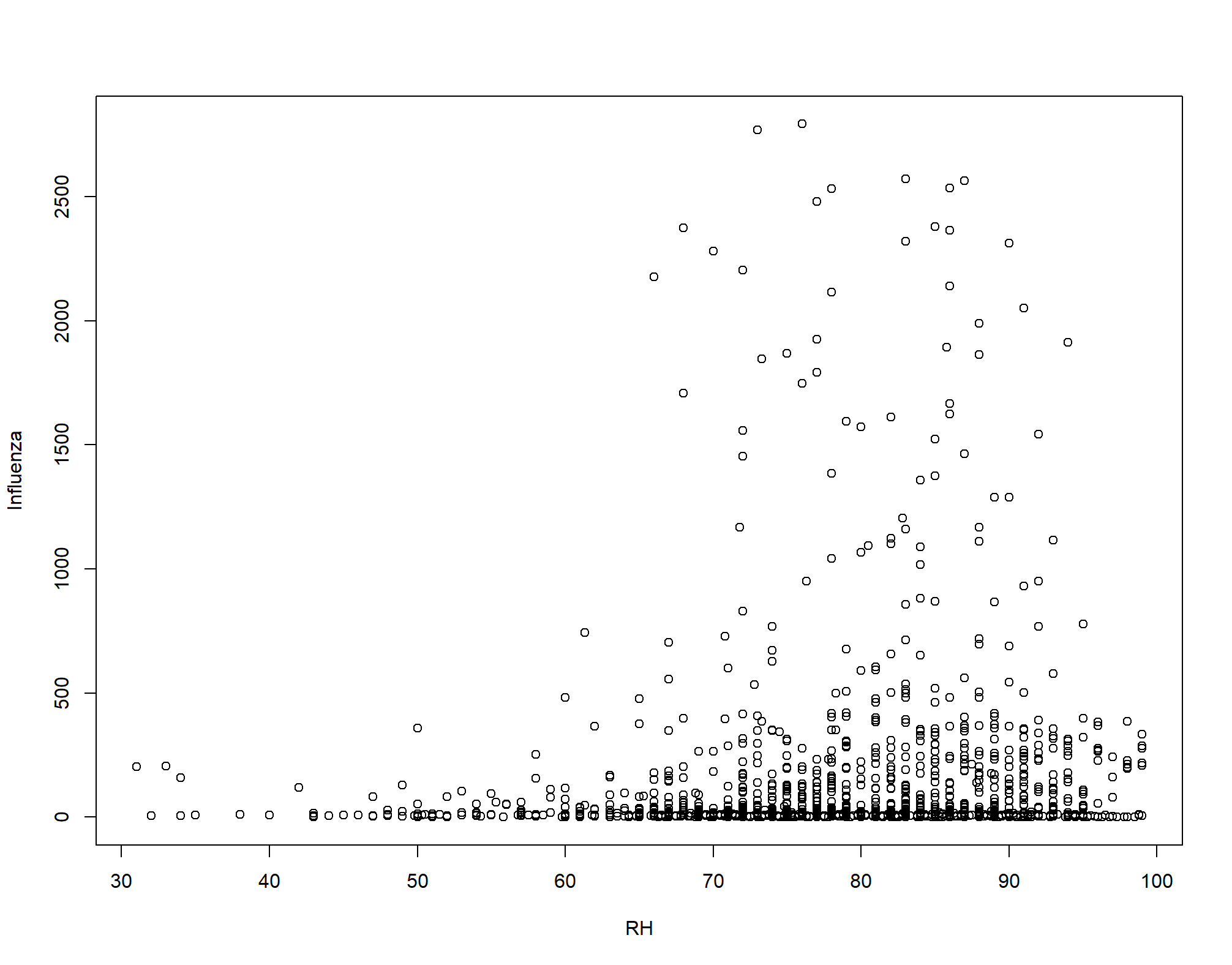


**Figure S2. The estimated overall effects of mean temperature on influenza cases by gender and age.**


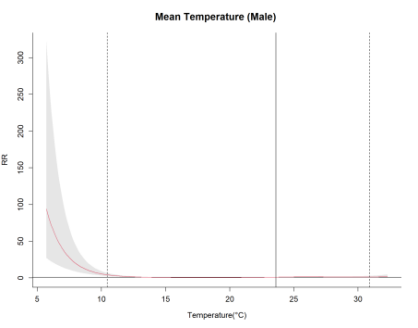

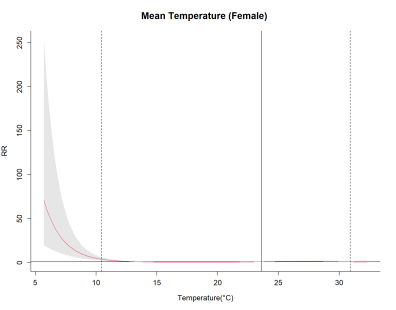

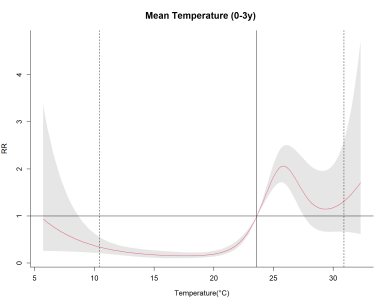

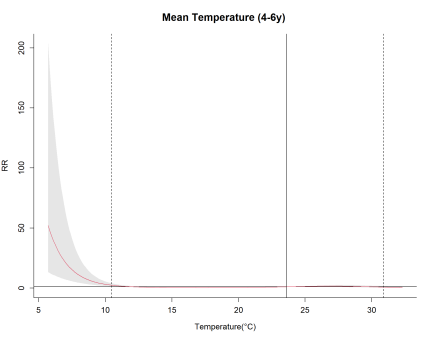

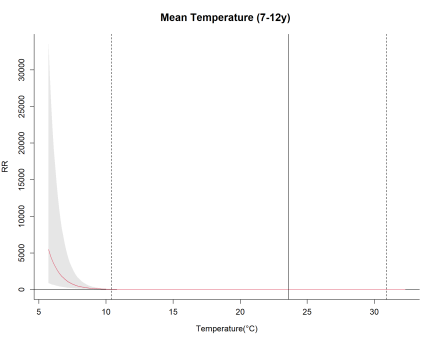

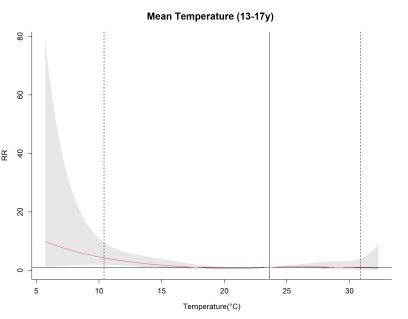


**Figure S3. The estimated overall effects of relative humidity on influenza cases by gender and age.**


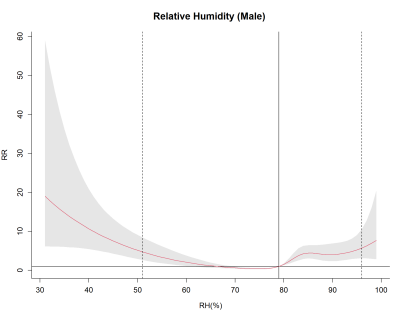

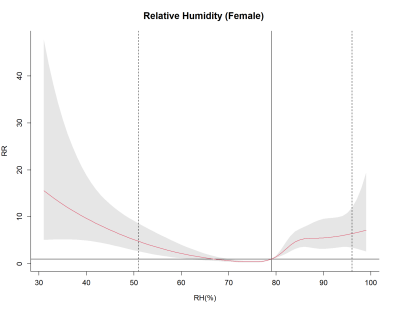

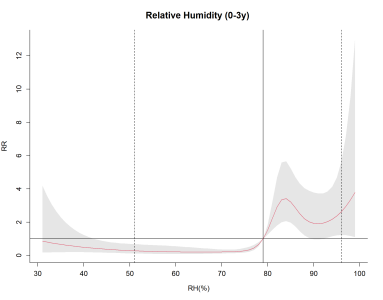


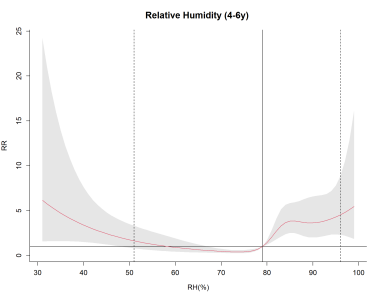

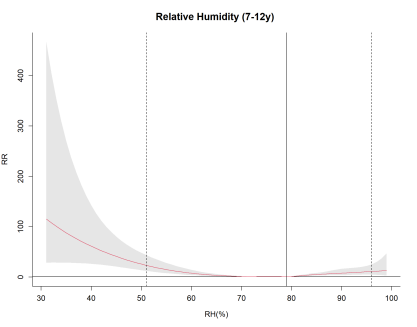

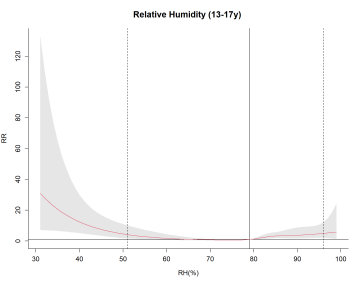


**Figure S4. The estimated overall effects of rainfall on influenza cases by gender and age.**


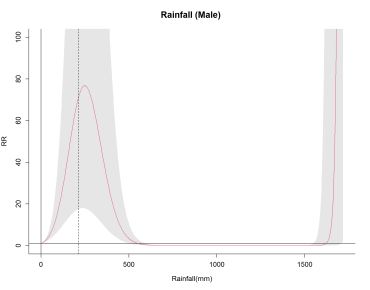

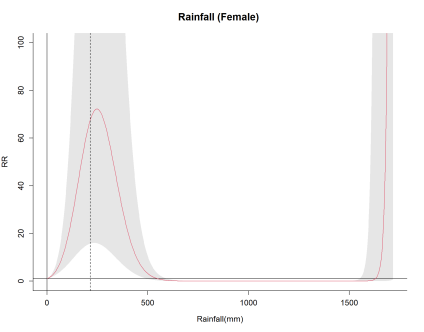

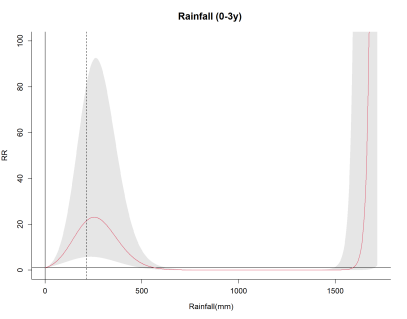


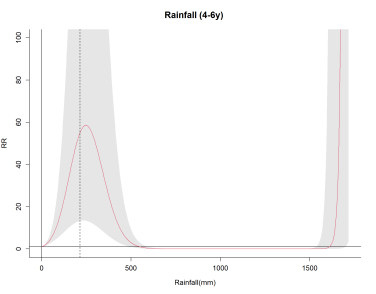

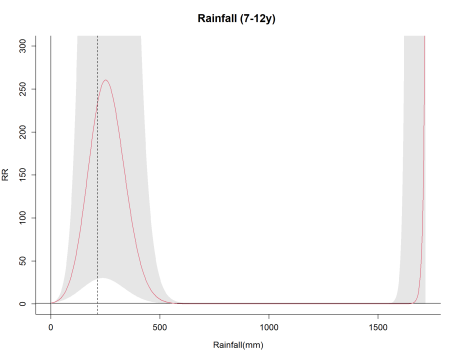

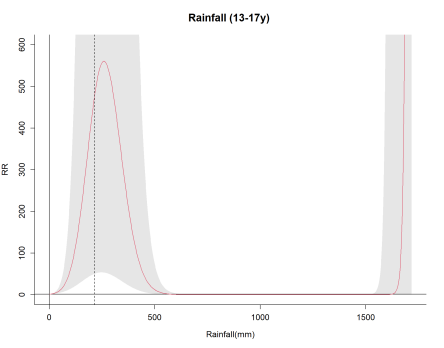


**Figure S5. The estimated overall effects of sunshine hours on influenza cases by gender and age.**


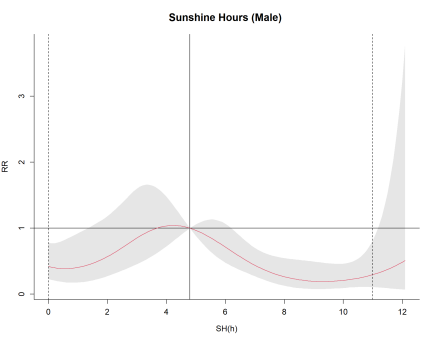

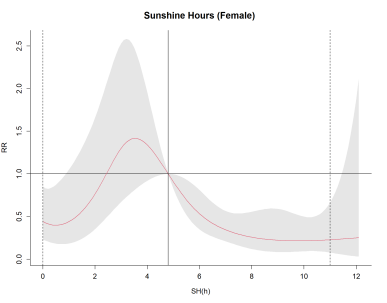

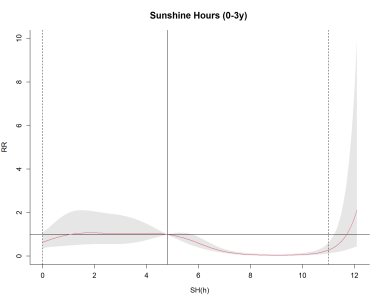


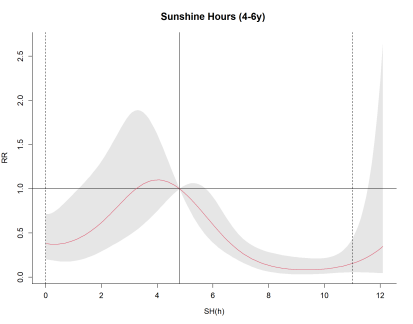

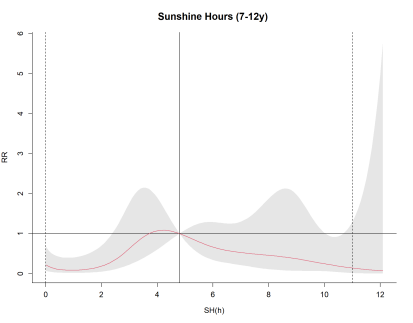

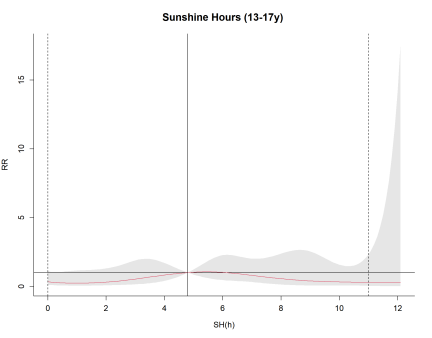


| **Table S1. The Akaike Information Criterion (AIC) values by changing the degrees of freedom (df) for “time”.** | | | | |
| --- | --- | --- | --- | --- |
|  | df | | | |
|  | 4 | 5 | 6 | 7 |
| time | 72135.71 | 79048.3 | **36993.37** | 37402.19 |
| Bold represented the optimal parameter. | | | | |

| **Table S2. The Akaike Information Criterion (AIC) values by changing the maximum lag weeks and degrees of freedom (df) for meteorological factors.** | | | | | |
| --- | --- | --- | --- | --- | --- |
| Variables | Maximum lag (weeks) | df | | | |
|  |  | 2 | 3 | 4 | 5 |
| Tmean | 2 | 32257.49 | 29546.86 | 28839.33 | 28756.73 |
|  | 3 | 29376.76 | 27096.74 | 26173.68 | 26120.47 |
|  | 4 | 28642.53 | 25963.28 | 24309.48 | **24089.69** |
| RH | 2 | 29099.83 | 28245.79 | 27887.45 | 27875.74 |
|  | 3 | 30205.74 | 27688.58 | 26720.69 | 25919.41 |
|  | 4 | 29234.98 | 27697.89 | 26012.62 | **25079.87** |
| Rainfall | 2 | / | 33074.49 | / | / |
|  | 3 | / | 32398.79 | / | / |
|  | 4 | / | **32328.41** | / | **/** |
| SH | 2 | 29466.36 | 29150.69 | 29214.21 | 29103.91 |
|  | 3 | 29441.19 | 28541.01 | 28420.48 | 28171.72 |
|  | 4 | 28872.04 | 28233.42 | 27541.30 | **27000.98** |
| Bold represented the optimal parameter. Changing the degree of freedom for rainfall would lead to errors in the model, only the lag weeks were changed. | | | | | |

| **Table S3. The extreme effects of meteorological variables on influenza cases of children along the lag weeks in Guangzhou, China, 2019-2022.** | | | | | | | |
| --- | --- | --- | --- | --- | --- | --- | --- |
| **Variables** | **Values** | **Relative Risks (95%CI)** | | | | | |
|  |  | **lag 0** | **lag 1w** | **lag 2w** | **lag 3w** | **lag 4w** | **lag 0-4w** |
| **T mean** | 10.4(°C) | 0.87^*^  (0.82,0.93) | 1.07^*^  (1.04,1.10) | 1.06^*^  (1.03,1.09) | 1.11^*^  (1.08,1.14) | 1.05  (0.99,1.11) | 4.23^*^  (2.67,6.70) |
|  | 30.9(°C) | 0.97  (0.91,1.04) | 0.95^*^  (0.91,0.99) | 0.96  (0.92,1.01) | 1.09^*^  (1.04,1.14) | 1.06  (0.97,1.15) | 1.10  (0.52,2.36) |
| **RH** | 51(%) | 0.94  (0.87,1.01) | 1.10^*^  (1.05,1.14) | 1.07^*^  (1.03,1.11) | 1.06^*^  (1.02,1.10) | 1.04  (0.98,1.11) | 4.76^*^  (2.74,8.26) |
|  | 96(%) | 0.94  (0.89,1.00) | 1.11^*^  (1.08,1.14) | 1.07^*^  (1.04,1.10) | 1.06^*^  (1.03,1.09) | 1.08^*^  (1.04,1.13) | 6.04^*^  (3.36,10.85) |
| **Rainfall** | / | / | / | / | / | / | / |
|  | 215  (mm) | 1.09  (0.99,1.21) | 1.45^*^  (1.34,1.57) | 1.19^*^  (1.11,1.27) | 0.98  (0.92,1.05) | 1.00  (0.92,1.08) | 70.21^*^  (17.75,277.67) |
| **SH** | 0.0(h) | 0.95  (0.90,1.00) | 1.01  (0.98,1.04) | 0.96^*^  (0.93,0.99) | 0.95^*^  (0.92,0.98) | 0.98  (0.94,1.03) | 0.43^*^  (0.23,0.79) |
|  | 11.0(h) | 0.99  (0.91,1.06) | 0.96  (0.91,1.02) | 0.97  (0.91,1.02) | 0.89^*^  (0.84,0.94) | 1.05  (0.98,1.13) | 0.27^*^  (0.10,0.72) |
| *: the cumulative extreme effects of meteorological variables on influenza cases of children are significant at 95% confidence intervals. Since both the median and the 2.5^th^ percentile of rainfall were 0, the extreme low effect could not be calculated. T mean: mean temperature; RH: mean relative humidity; Rainfall: aggregate rainfall; SH: sunshine hours. | | | | | | | |
